# Soil and Rhizosphere Associated Fungi in Gray Mangroves (*Avicennia marina*) from the Red Sea — A Metagenomic Approach

**DOI:** 10.1016/j.gpb.2015.07.002

**Published:** 2015-11-05

**Authors:** Marta Filipa Simões, André Antunes, Cristiane A. Ottoni, Mohammad Shoaib Amini, Intikhab Alam, Hanin Alzubaidy, Noor-Azlin Mokhtar, John A.C. Archer, Vladimir B. Bajic

**Affiliations:** 1 Computational Bioscience Research Center (CBRC), Computer, Electrical and Mathematical Sciences and Engineering Division (CEMSE), King Abdullah University of Science and Technology (KAUST), Thuwal 23955-6900, Saudi Arabia; 2 Laboratório de Biotecnologia Industrial (LBI), Instituto de Pesquisas Tecnológicas do Estado de São Paulo (IPT), São Paulo 05508-901, Brazil

**Keywords:** Fungal diversity, Ascomycota, Basidiomycota, Red Sea, Metagenomics, Bioinformatics

## Abstract

Covering a quarter of the world’s tropical coastlines and being one of the most threatened ecosystems, mangroves are among the major sources of terrestrial organic matter to oceans and harbor a wide microbial diversity. In order to protect, restore, and better understand these ecosystems, researchers have extensively studied their microbiology, yet few surveys have focused on their fungal communities. Our lack of knowledge is even more pronounced for specific fungal populations, such as the ones associated with the rhizosphere. Likewise, the **Red Sea** gray mangroves (*Avicennia marina*) remain poorly characterized, and understanding of their fungal communities still relies on cultivation-dependent methods. In this study, we analyzed **metagenomic** datasets from gray mangrove rhizosphere and bulk soil samples collected in the **Red Sea** coast, to obtain a snapshot of their fungal communities. Our data indicated that **Ascomycota** was the dominant phylum (76%–85%), while **Basidiomycota** was less abundant (14%–24%), yet present in higher numbers than usually reported for such environments. Fungal communities were more stable within the rhizosphere than within the bulk soil, both at class and genus level. This finding is consistent with the intrinsic patchiness in soil sediments and with the selection of specific microbial communities by plant roots. Our study indicates the presence of several species on this mycobiome that were not previously reported as mangrove-associated. In particular, we detected representatives of several commercially-used fungi, *e.g.*, producers of secreted cellulases and anaerobic producers of cellulosomes. These results represent additional insights into the fungal community of the gray mangroves of the **Red Sea**, and show that they are significantly richer than previously reported.

## Introduction

Mangroves are endangered coastal biotopes that approximately cover a quarter of the world’s tropical coastlines [1], [2], [3]. They are associated with a wide range of ecological benefits, such as being a major source of terrestrial organic matter to oceans and are well recognized, yet poorly studied, biodiversity hotspots [2], [4]. Microbes are major components of this biodiversity, with bacteria and fungi constituting 91% of the total biomass of mangrove ecosystems [5], with the fungal fraction being the least studied.

Fungi are a ubiquitous and very diverse group of organisms currently comprising seven recognized phyla: Basidiomycota, Ascomycota, Glomeromycota, Microsporidia, Blastocladiomycota, Neocallimastigomycota, and Chytridiomycota [6]. Generally, fungi are important soil components as both decomposers and plant symbionts, playing major roles in ecological and biogeochemical processes [7]. They contribute significantly to the degradation of mangrove-derived organic matter [8], being its primary mineralizers in mangrove sediments and representing important food source for benthic fauna [2].

Fungal surveys in mangroves have focused mainly on taxonomic diversity of saprophytic fungi retrieved from intertidal, floating or immersed, pieces of trees and wood debris [9]. Diversity estimates pointed to 625 marine fungi species associated with mangrove forests, and 269 related to mangrove roots [10]. These mangrove fungi are almost exclusively saprophytic and belong primarily to the Ascomycota (*e.g.*, sac fungi and yeasts) and Basidiomycota (*e.g.*, mushrooms, rusts, and smuts), which are members of the subkingdom Dikarya [6], [11].

A few studies analyzed mangrove-associated fungi [1], [8], [9], [12], [13]. Highest counts are often found in soil surfaces or in roots and rhizomes, and some studies related their growth peak with higher humidity [14], [15]. Unfortunately, information on fungal diversity in mangrove rhizospheres, the soil zone located in and around the active roots, is lacking and is mostly based on culture-dependent assessments [16], [17], [18]. As is well known and widely reported, traditional culturing techniques only succeed in isolating a very limited percentage of microorganisms and fail to capture the full microbial diversity present in the environment [19]. Previous reports pointed out that from the total (under)estimated 1.5 × 10^6^ fungal species, only *ca*. 8%–10% have been identified [6]. Culture-independent techniques, *e.g.*, metagenomics, successfully circumvent such culture-based biases [7], [20], [21] and are essential for studying the real fungal diversity present in mangroves [1], [4], [8].

Contrasting with other seas, the Red Sea exhibits an antagonistic salinity-temperature profile: moving from south to north, surface water temperature decreases from 33.8 °C down to 21 °C; and, salinity increases from 37 to 41 Practical Salinity Unit (PSU) [22]. Such salinities, which are higher than the world average, are further increased in mangrove shallow waters [9], [22]. The high levels of stress imposed on the mangroves of the Red Sea result in scattered forests, decreased floral diversity, and limited plant height [23], [24], [25], [26]. Moreover, mangroves in the northern coastline of the Red Sea are mono-specific, and composed exclusively of *Acivennia marina* (gray mangrove) [9].

Information on fungal diversity in the gray mangroves of the Red Sea is scarce. In one of the very few studies available, Abdel-Wahad et al. [9] used a targeted metagenomic approach to look into fungal diversity of the soil and rhizosphere in gray mangroves from the Red Sea. They recorded a total of 29 different fungal species isolated from wood pieces on the mangroves and surrounding beaches, although the rhizosphere remained under-studied.

In order to decrease the paucity of data on fungal communities present in rhizosphere and in the gray mangroves of the Red Sea, we analyzed samples collected from this specific environment. Our results are a valuable addition that further clarifies our understanding of these communities.

## Results and discussion

### Eukaryotic and fungal representation within the soil and rhizosphere samples

Studies of four metagenomic samples from sediments of gray mangrove rhizosphere (RSMgr 01–04) and two samples from bulk soil (CS 01 and CS 02), publicly available under the project name “*A. marina* rhizosphere”, were retrieved and analyzed at the metagenomics analyzer server (http://metagenomics.anl.gov). These metagenomic datasets from gray mangroves of the Red Sea revealed that Eukaryota represent a relatively small percentage of all reads. Total number of eukaryotic reads slightly increased from control samples (bulk soil) to rhizosphere sediments, (0.6%–0.7% and 2% of total reads, respectively), while fungal abundance was much higher in the rhizosphere sediments (Table 1). Such low abundances occur despite the widely-recognized importance of mangrove fungi, and the fact that they represent the second major ecological group of marine fungi (*e.g.*, [27], [28]). We should note that Kuramae et al. [29] showed that fungal abundance is significantly correlated with phosphate, while frequent water logging and subsequent episodic anaerobic conditions were proposed as the possible explanations for the low abundance of fungi in some soils. Furthermore, it has been previously reported that the fungal abundance is lower in mangroves with smaller stands and tree-size, as well as less diverse regarding tree flora [30]. The mangroves of the northern Red Sea show all of these features. Despite being very rich in carbon (C), mangrove soils are frequently nutrient-poor, with extremely low nutrient availability [31]. Mangroves have evolved in tropical oligotrophic tidal environments with their soils having characteristically very low contents of total nitrogen (N) and phosphorous (P) [31]. Such an effect is even more pronounced in the ultra-oligotrophic environment of the Red Sea [32]. Consequently, mangrove forests in the north of the Red Sea are sparse, with trees displaying decreased height and appearing in patchy and scattered patterns [23], [24], [25], [26].

**Figure 1:**
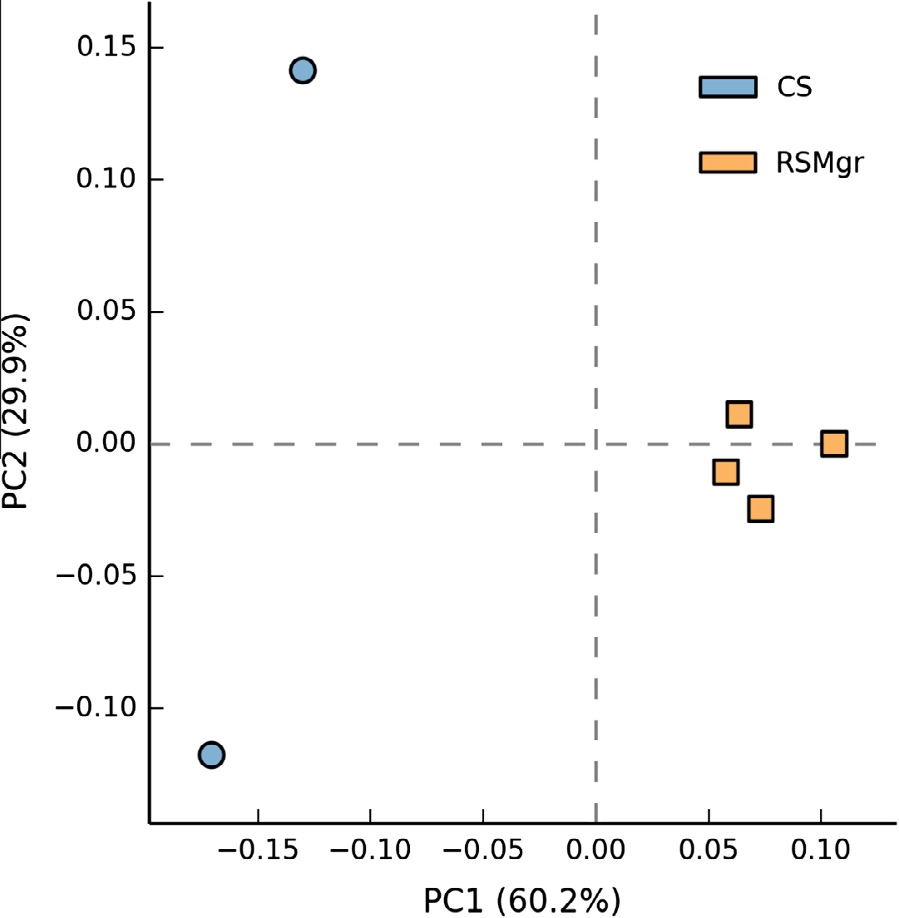
**Principal components analysis of the fungal communities in the Red Sea gray mangrove samples** Analysis was performed based on read counts at class level. CS represents bulk soil samples and RSMgr represents gray mangrove rhizosphere samples. PC1, first principal component, represents 60.2% of the variation in data; PC2, the second principal component, represents 29.9% of the variation in data.

In order to confirm and better represent the fungal diversity differences between CS and RSMgr samples, we performed principal component analysis (PCA) as described in Materials and methods section with read counts at class level, which provides enough analysis power. Figure 1 shows the CS and RSMgr samples at the class level in the plane with axes as first and second principal components, respectively. We observed that at the class level, the fungal communities of CS samples were distinct from those of RSMgr samples, with the latter displaying lower intra-group variability. In fact, this is also evident even with the first principal component (PC1) values.

### Fungal abundance analysis at phylum level

**Figure 2:**
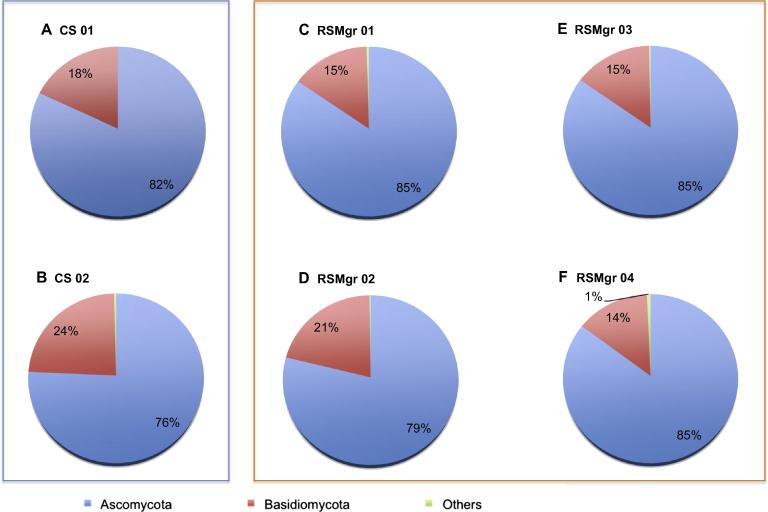
**Eukaryota distribution in different samples from Red Sea gray mangroves** The read proportion of Eukaryota distribution in different samples. CS, bulk soil; RSMgr, gray mangrove rhizosphere. “Others” include Neocallimastigomycota, Blastocladiomycota, Glomeromycota, and Chytridiomycota.

In contrast to the aforementioned low abundances of Eukaryota in soil and rhizosphere samples, we detected a very high fungal abundance, particularly pronounced for the rhizosphere samples (Table 1). At the phylum level (Figure 2), fungal communities were clearly dominated by Ascomycota (76%–85%) and Basidiomycota (14%–24%). Members of these two phyla are expected to play an important ecological function in the mangroves [1]. Ascomycetes from marine environments are an important ecological assembly of mostly saprophytic microbes occurring in different substrata rich in lignin, cellulose, or chitin [33]. Other trophic levels are dependent on the lignocellulose-cleaving capability of these fungi that allow this complex substrate to enter the food web [33]. Basidiomycetes are also mostly saprophytes [1], yet are mostly excluded from aquatic environments, leading to lower abundances [3]. Other previous studies of soils [8], [14], [34], marine environments [35], and mangroves in general [9], [10], [12], [36] pointed similarly to a predominance of Ascomycota and Basidiomycota. Nevertheless, we have found Basidiomycota to be more frequent here than has been described for other mangrove associated fungal communities (*e.g.*, [1], [8], [12], [37]).

A study by Lauber et al. [38] showed that the fluctuations in relative abundance of Ascomycota and Basidiomycota in different types of soil were attributable to variations in C/N ratios and levels of P. Succinctly, P-rich soils contain more Ascomycota, and fewer Basidiomycota, while soils with higher C/N ratios have a higher prevalence of Basidiomycota. These findings are in agreement with our results. RSMgr 01, which possessed the highest P concentration in combination with lowest C/N ratio (considering organic matter and nitrate as proxies for C and N, Table S1), had the highest number of reads for Ascomycota (85%) and the lowest for Basidiomycota (15%), while opposite nutrient distribution was observed for CS 02, which had lower P content and a higher C/N ratio (Figure 2 and Table S1). Interestingly, CS 02 had the highest relative abundance of Basidiomycota (76% Ascomycota and 24% Basidiomycota).

### Fungal abundance analysis at class level

**Figure 3:**
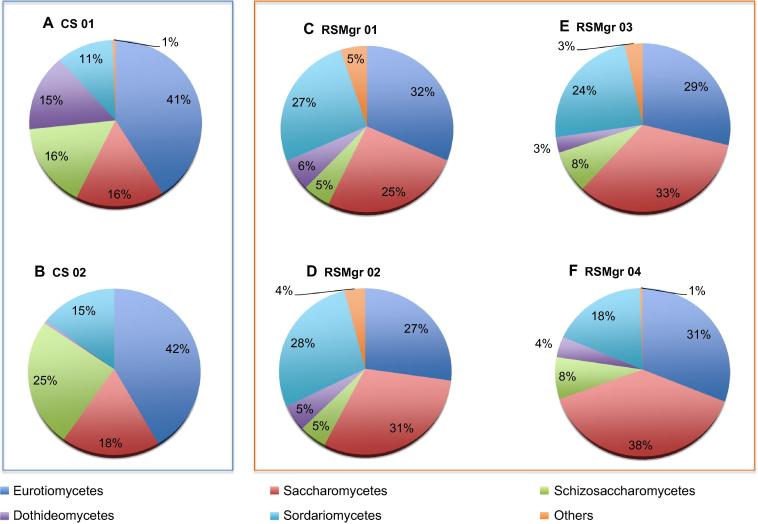
**Ascomycota distribution in different samples from Red Sea gray mangroves** The read proportion of Ascomycota distribution in different samples. CS, bulk soil; and RSMgr, gray mangrove rhizosphere. “Others” include Leotiomycetes, Pneumocystidomycetes, Lecanoromycetes, Orbiliomycetes, and Pezizomycetes.

**Figure 4:**
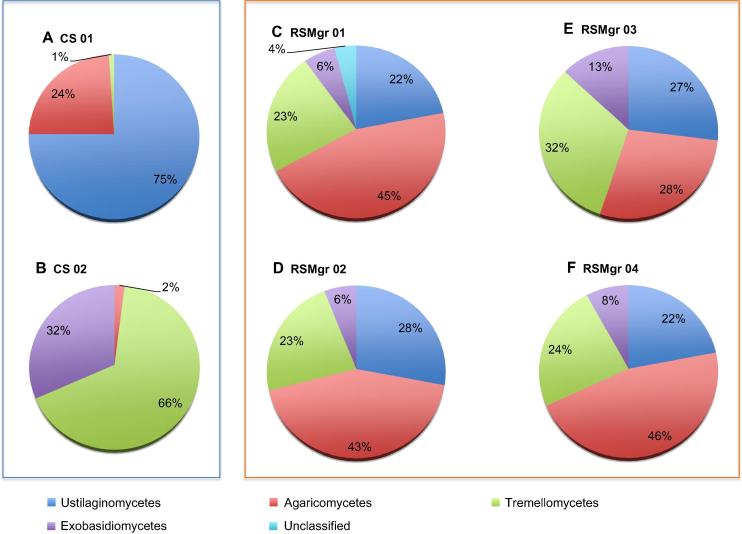
**Basidiomycota distribution in different samples from Red Sea gray mangroves** The read proportion of Basidiomycota distribution in different samples. CS, bulk soil; RSMgr, gray mangrove rhizosphere. “Unclassified” are derived from Basidiomycota.

Despite being mostly similar at the phylum level, we noted significant differences at the class level among the samples from the soil (CS) and the rhizosphere (RSMgr), in both Ascomycota (Figure 3) and Basidiomycota (Figure 4). Within the phylum Ascomycota, CS 01 and CS 02 contained comparable percentage of the class Eurotiomycetes (41% and 42%), which is higher than that found in the RSMgr samples (27%–31%). Similarly, more Schizosaccharomycetes were also found in the CS samples (16% and 25%) in comparison to the RSMgr samples (5%–8%).

**Figure 5:**
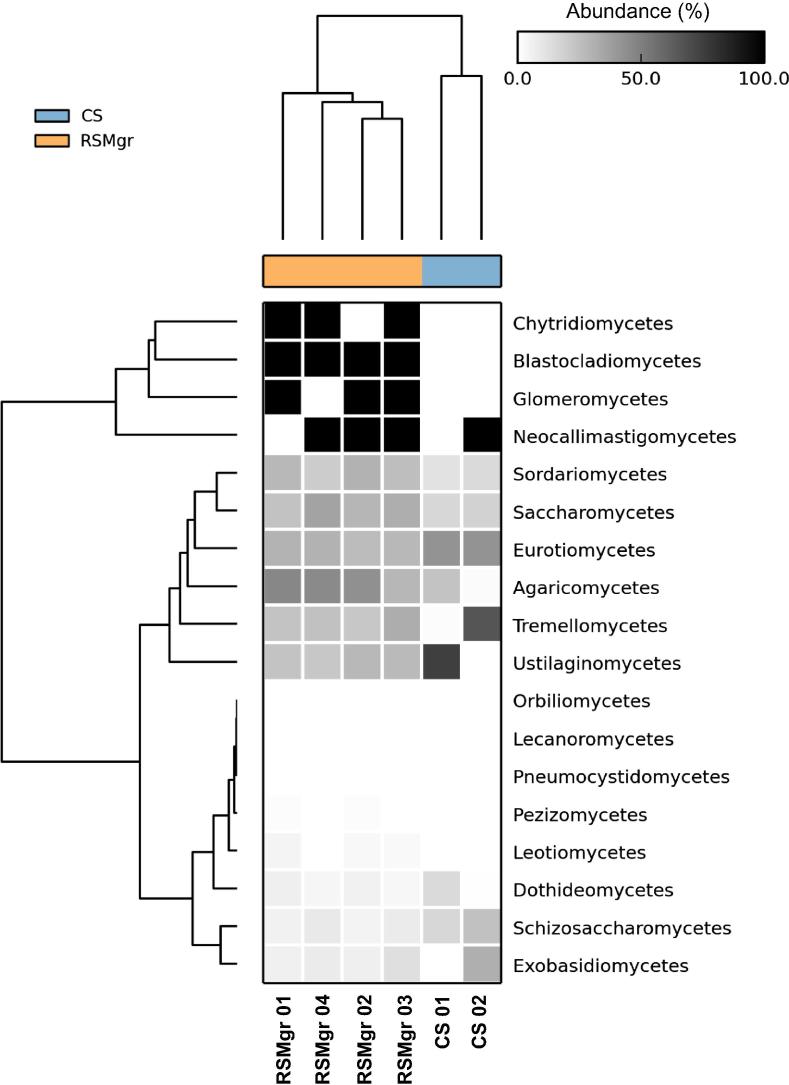
**Heat map of the relative abundances of the fungal communities in Red Sea gray mangroves samples** Heat map of the relative abundances of the fungal communities generated with read counts by using STAMP software. CS, bulk soil; RSMgr, gray mangrove rhizosphere.

**Figure 6:**
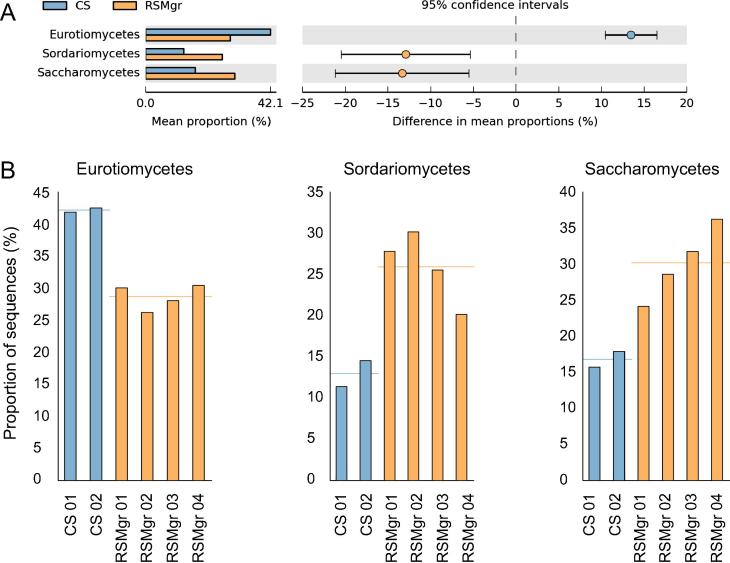
**Read percentage of Eurotiomycetes, Sordariomycetes, and Saccharomycetes in CS and RSMgr samples** Read count of the three most statistically relevant classes, Eurotiomycetes, Sordariomycetes, and Saccharomycetes varied in CS and RSMgr samples. **A.** Comparison of the mean proportions of the three classes. **B.** Individual bar plot of the three classes. CS represents bulk soil and is marked in blue, whereas RSMgr represents gray mangrove rhizosphere and is marked in orange. The comparisons were made between the group of CS and the group of RSMgr samples (*P *= 3.90E−4 for Eurotiomycetes, 9.45E−3 for Sordariomycetes, and 9.56E−3 for Saccharomycetes, respectively).

When comparing the fungal communities across all six samples (Figure 5), we noted that samples can be grouped according to the diversity present. CS samples group apart from the RSMgr samples, while RSMgr 03 and RSMgr 04 show more similar diversities. But the most significant differences were found in the increased percentage of Saccharomycetes (25%–38% *vs.* 16%–18%; *P *= 9.56E−3) and Sordariomycetes (18%–28% *vs.* 11%–15%; *P *= 9.45E−3) in the RSMgr samples when compared to CS samples but decreased content of Eurotiomycetes (26%–31% *vs.* 41%–42%; *P *= 3.90E−4), as shown in Figure 6.

Class Dothideomycetes accounted for 3%–6% of Ascomycota in RSMgr samples (Figure 3). However, high variability was found in the percentage of class Dothideomycetes in the CS samples (15% for CS 01 and 0.6% for CS 02). Even though rhizosphere samples show more class variability, this was not the case for Dothideomycetes.

Within the phylum Basidiomycota (Figure 4), all the RSMgr samples possessed similar percentage of Ustilaginomycetes (22%–28%). However, RSMgr 01, RSMgr 02, and RSMgr 04 had similar content of Agaricomycetes (43%–46%), Tremellomycetes (23%–24%) and Exobasidiomycetes (6%–8%), whereas RSMgr 03 had much lower percentage of Agaricomycetes (28%) but more Tremellomycetes (32%) and Exobasidiomycetes (13%). On the other hand, CS samples appeared to have a very different composition of Basidiomycota: 75% of Ustilaginomycetes and 24% Agaricomycetes in CS 01; 67% of Tremellomycetes and 32% of Exobasidiomycetes in CS 02).

### Fungal abundance analysis at genus and species level

Overall, we found that relative fungal diversity is more stable within the RSMgr than within the CS. Such results are consistent with the intrinsic environmental and biological patchiness in soil sediments, and with the preferential selection of specific microbial communities by plant roots.

A similar trend was observed at the genus level, with identical genera listed in the top ten for RSMgr, and very different profiles for CS (Table 2). It was noticeable that the genus *Aspergillus* and *Schizosaccharomyces* clearly dominate in all the samples examined in this study. Both genera are known producers and secretors of a large variety of heterologous proteins [39]. For example, *Aspergillus oryzae* [40] and *Schizosaccharomyces pombe* [41], the well-known and well-studied examples of heterologous proteins producers, were identified in large amounts and in most of these samples for both bulk and rhizosphere soil (Table S2). Dominance of *Aspergillus* is in accordance with previous studies [42] reporting that this genus is frequently found in marine sediments. Such dominance also agrees with the isolations made by Thamizhmani and Senthilkumaran [43] from mangrove sediments, where they found several different species of this genus. In addition to *Aspergillus*, they also identified *Emericella* and *Neurospora* in their samples, which were also found in our samples, although the abundance of *Emericella* was low.

From the metadata hereby presented, a total of 145 different species within 109 different genera were identified (Table S2). Our results bring to light the existence of many additional species on the mycobiome of Red Sea mangrove rhizosphere that were not previously reported as mangrove-associated, since most prior information is based on culture-dependent research. Furthermore, we anticipate total fungal diversity in the gray mangroves of the Red Sea to be even higher than shown by our study. It is well known that geochemical parameters (*e.g.*, salinity, soil humidity, and nutrients concentrations) for mangroves vary cyclically, throughout the day, with tides, and with seasons [1]. To fully capture total fungal diversity, additional sampling at different times and locations is essential while taking these variations into account.

Bioprospecting for potentially biotechnological interesting microbes is one of the many utilities that derive from characterizing microbial communities. This task has been largely improved by the use of culture-independent techniques [21]. Metagenomic analysis of fungal diversity of rhizospheres and sediments of mangroves would facilitate the discovery of novel enzymes, bioactivities, and relevant secondary metabolites. Mangroves are a source of cellulosic substrates and are at the transition between terrestrial and aquatic environment. They are a dynamic ecotone subjected to harsh conditions, with fluctuating temperature, salinity, and tides [3]. These environmental characteristics lead to fungal community specialized in producing a wide array of hydrolytic enzymes such as cellulases [10]. This class of enzymes is under intense study for their involvement in biofuel production from renewable cellulosic substrates [19], as favorite candidates for industrial and biotechnology applications [10]. Sahoo et al. [44] noted that mangrove soil is a good source of microbes able to degrade polythene and plastics. In our samples, we found representatives of commercially-used producers of secreted cellulases: *Aspergillus*, *Fusarium, Phanerochaete*, and *Penicillium*; and also anaerobic fungi producers of cellulosomes, complexes of cellulases with high molecular weight: *Orpinomyces*, *Piromyces*, and *Neocallimastix* [10].

### Fungal diversity analyses

Alpha-diversities for the total amount of reads were obtained using the Metagenomic Rapid Annotations with Subsystems Pipeline (MG-RAST) pipeline, as a means to further quantify fungal diversity of the annotated samples. The following evaluation using the species-level annotation distribution showed higher total species diversity in CS samples (Table 3). However, as shown in the previous sections, fungal diversity revealed higher richness in RSMgr samples (Table 3). It is well known that as species richness and evenness (the measure of relative abundance of the different species) increase, so does diversity. Simpson index measures the probability that two individuals randomly selected from a sample belong to the same species, which relates richness with evenness of the population, with higher Simpson index suggesting lower diversity. As shown in Table 3, RSMgr samples had much lower Simpson indexes, thus demonstrating a higher fungal diversity when compared with CS samples.

### Overview and future work

Our study reveals that the diversity of fungal communities in the gray mangroves of the Rea Sea is significantly wider than previously reported. Future studies on fungal community characterizations and bioprospection are recommended for these particular environments (including more thorough sampling efforts), because there is a high probability of very interesting findings. Due to the unique environmental characteristics of the rhizosphere of mangroves, it represents a unique and under-explored source for a pool of uncommon fungi with particular features of relevance for biotechnology, science, and health research.

## Materials and methods

### Sample collection

Sample collection was performed from six different sites along a 978 m transect of mangrove shore in Thuwal, Saudi Arabia, in December 2011 [45]. Four samples from sediments of gray mangrove rhizosphere (RSMgr 01–04), and two samples from bulk soil (CS 01 and CS 02) as control were collected. It is important to note that, at each site, samples were collected from a 10-cm depth aseptically and stored at 4 °C prior to subsequent processing within 12 h. Chemical analyses for each sample were performed as follows. Briefly, phosphorous concentrations were measured with microwave-assisted digestion method [45]. Nitrate content was measured directly using Autoanalyzer/Photometric Analyzer, Aquakem250 (Thermo Scientific, Vantaa, Finland). Content of organic matter was calculated with the loss on ignition method [45], [46], which was 9.21%−10.12% for RSMgr samples and 2.53%−3.19% for CS samples, respectively. The temperature and salinities were measured with a 5 Star pH/ISE/ORP/DO Conductivity Portable Meter (Thermo Fisher Scientific, Waltham, Massachusetts, USA). The temperature was 21.2 °C for all the samples. The salinities of the RSMgr samples were 18.65–23.38 PSU, whereas CS samples had salinities of 8.40–14.23 PSU.

### Dataset acquisition

Metagenomes were obtained through DNA extraction of each sample using the ZR Soil Microbe DNA MidiPrep kit (Zymo Research, Irvine, CA) and pyrosequencing with 454 GS FLX Titanium (Roche Applied Science, Indianapolis, Indiana, USA) in the Bioscience Core Laboratory, King Abdullah University of Science and Technology (Thuwal, Saudi Arabia). These data, publicly available on http://metagenomics.anl.gov/, under the project name “*A. marina* rhizosphere”, detailed on Table S3, were retrieved and analyzed through the MG-RAST [47] at the metagenomics analyzer server.

We compared the data to M5NR using the maximum cutoff *E*-value of 1E−5; we used 60% as the minimum identity cutoff, and the minimum alignment length cutoff of 15, measured in base pairs for RNA databases and in amino acids for proteins.

### Statistical analyses

The similarity among the collected samples was analyzed with principal component analysis (PCA), based on relative abundance at class level using domain as the parent level.

Alpha-diversities, the number of distinct species in a given sample, were achieved by the distribution of the species-level annotations (total species from all taxonomic domains) obtained from MG-RAST.

Simpson index for the fungal population was calculated, using the formula:

$$D=\frac{\sum_{i}n_{i}(n_{i}-1)}{N(N-1)}$$

where *n_i_* represents the total number of organisms of a particular species and *N* represents the total number of organisms of all species [48].

Heat map was generated using the Statistical Analyses of Metagenomic Profiles (STAMP) software [49] for fungal relative abundances. The associated dendrograms were obtained using the Unweighted Pair Group Method with Arithmetic Mean (UPGMA) with a clustering threshold of 0.75.

Relevant differences in the relative proportions of the classified sequences and mean proportions of the most statistically-relevant classes found in the samples were detected after analyzing the MG-RAST taxonomic profiles with STAMP. Datasets were analyzed with the two-sided Welch’s test, and we removed all unclassified reads from the analysis. *P* values of 0.05 were used as a filter to determine the most important taxa, and we only used those categories with more than 2-fold ratio between the proportions and with difference between the proportions of at least 1%.

## Authors’ contributions

MFS conceived, designed and performed the assays. MFS, AA, and CO analyzed the data. MSA, IA, HA, NAM, and JACA contributed with reagents/materials/analysis tools. MSA performed the statistical analyses. MFS, AA, and CO drafted the manuscript with the help of MSA, IA, and VBB. All authors read and approved the final manuscript.

## Competing interests

The authors declare no competing interests.

## Figures and Tables

**Table 1:** Sequencing reads for Eukaryota and fungi in different samples

**Sample**	**Total No. of raw reads**	**Eukaryota from all domains (%)**	**Fungal abundance**		**No. of fungal genera**
**No. of reads**	**%**
CS 01	705,326	0.6	370	0.05	18
CS 02	514,784	0.7	237	0.05	10
RSMgr 01	1,267,409	2	1771	0.14	71
RSMgr 02	1,416,928	2	2047	0.14	52
RSMgr 03	854,451	2	1828	0.21	55
RSMgr 04	1,045,353	2	2020	0.19	50

**Table 2:** Top 10 abundant genera found in each sample

**Soil samples**				**Rhizosphere samples**							
**CS 01**		**CS 02**		**RSMgr 01**		**RSMgr 02**		**RSMgr 03**		**RSMgr 04**	
**Genus**	**%**	**Genus**	**%**	**Genus**	**%**	**Genus**	**%**	**Genus**	**%**	**Genus**	**%**
*Aspergillus*	24.1	*Aspergillus*	32.1	*Aspergillus*	12.3	*Aspergillus*	10.2	*Aspergillus*	13.1	*Aspergillus*	13.3
*Ustilago*	13.8	*Schizosaccharomyces*	18.6	*Gibberella*	5.8	*Neurospora*	5.9	*Schizosaccharomyces*	6.5	*Gibberella*	6.8
*Schizosaccharomyces*	12.7	*Filobasidiella*	16.0	*Schizosaccharomyces*	4.6	*Gibberella*	5.4	*Neurospora*	5.7	*Schizosaccharomyces*	6.3
*Phaeosphaeria*	11.6	*Podospora*	11.0	*Neurospora*	3.9	*Magnaporthe*	4.2	*Gibberella*	5.3	*Neurospora*	5.4
*Chaetomium*	9.2	*Kluyveromyces*	10.1	*Filobasidiella*	3.3	*Saccharomyces*	4.2	*Saccharomyces*	4.8	*Filobasidiella*	5.0
*Neosartorya*	8.9	*Malassezia*	7.6	*Ustilago*	3.3	*Ustilago*	4.1	*Filobasidiella*	4.5	*Penicillium*	5.0
*Nakaseomyces*	5.4	*Saccharomyces*	3.4	*Saccharomyces*	3.0	*Schizosaccharomyces*	4.0	*Penicillium*	4.5	*Saccharomyces*	4.9
*Kluyveromyces*	4.9	*Agaricus*	0.4	*Penicillium*	2.9	*Penicillium*	3.6	*Ustilago*	3.8	*Ustilago*	4.5
*Postia*	4.3	*Alternaria*	0.4	*Magnaporthe*	2.6	*Yarrowia*	3.5	*Yarrowia*	3.4	*Yarrowia*	3.7
*Saccharomyces*	2.4	*Piromyces*	0.4	*Yarrowia*	2.5	*Filobasidiella*	3.3	*Debaryomyces*	3.2	*Debaryomyces*	3.3

*Note:* The relative abundance of each genus is indicated as the percentage of reads per genus.

**Table 3:** Species diversity in the different samples

	**Samples**					
	**CS 01**	**CS 02**	**RSMgr 01**	**RSMgr 02**	**RSMgr 03**	**RSMgr 04**
Average length (bp)	256	255	566	563	558	542
Alpha-diversity (species level for all taxonomic domains)	869.90	889.88	580.16	603.71	776.61	704.71
No. of hits for fungi	370	237	1771	2047	1828	2020
Richness (No. of different species of fungi)	19	12	91	58	69	63
Simpson-index (for fungi species)	0.1246	0.1468	0.02128	0.03016	0.02946	0.03524

*Note:* Alpha-diversities were readouts from the Metagenomic Rapid Annotations with Subsystems Pipeline (MG-RAST).

## Supplementary material

Supplementary Table S1 Selected chemical parameters of the sampling locations in this study

Supplementary Table S2 Fungal species found in the different samples

Supplementary Table S3 Details for the dataset used in this study
