# Supplementary material for: Soil and Rhizosphere Associated Fungi in Gray Mangroves (Avicennia marina) from the Red Sea — A Metagenomic Approach
Source: Genomics Proteomics Bioinformatics. 2015 Nov 5;13(5):310–20. doi: 10.1016/j.gpb.2015.07.002 (PMC4678792; doi:10.1016/j.gpb.2015.07.002)
Supplement: Supplementary Table S1 — Selected chemical parameters of the sampling locations in this study [file mmc1.docx]

**Table S1 Selected chemical parameters of the sampling locations in this study**

| **Sample** | **Phosphate (ppm)** | **NO_3_^-^ (mg/l)** | **Organic matter(%)** |
| --- | --- | --- | --- |
| CS 01 | 168 ± 0.020 | 1.253 | 3.19 |
| CS 02 | 186 ± 0.031 | 1.412 | 2.53 |
| RSMgr 01 | 220 ± 0.044 | 5.542 | 9.21 |
| RSMgr 02 | 215 ± 0.030 | 3.598 | 10.12 |
