# Supplementary material for: Soil and Rhizosphere Associated Fungi in Gray Mangroves (Avicennia marina) from the Red Sea — A Metagenomic Approach
Source: Genomics Proteomics Bioinformatics. 2015 Nov 5;13(5):310–20. doi: 10.1016/j.gpb.2015.07.002 (PMC4678792; doi:10.1016/j.gpb.2015.07.002)
Supplement: Supplementary Table S3 — Details for the dataset used in this study [file mmc3.docx]

**Table S3 Details for the datasets analyzed in this study**

| **Sample** | **MG-RAST metagenome reference No.** | **Latitude** | **Longitude** |
| --- | --- | --- | --- |
| Control 01 | 4506447.3 | 22.50916667 | 39.19472222 |
| Control 02 | 4506448.3 | 22.49777778 | 39.19472222 |
| RSMgr 01 | 4523017.3 | 22.31916667 | 39.20666667 |
| RSMgr 02 | 4523018.3 | 22.50055556 | 39.21166667 |
| RSMgr 03 | 4523019.3 | 22.34666667 | 39.20833333 |
| RSMgr 04 | 4523020.3 | 22.35361111 | 39.20805556 |

*Note*: All the datasets analyzed in this study were stored as the public project entitled “*Avicennia marina* rhizosphere” at http://metagenomics.anl.gov/.
